# LncRNA HCG11/miR‐26b‐5p/QKI5 feedback loop reversed high glucose‐induced proliferation and angiogenesis inhibition of HUVECs

**DOI:** 10.1111/jcmm.16040

**Published:** 2020-10-30

**Authors:** Jiao Du, Ruijuan Han, Yihua Li, Xiaolin Liu, Shurong Liu, Zhenyu Cai, Zhaolong Xu, Ya Li, Xuchun Yuan, Xiuhai Guo, Bin Lu, Kai Sun

**Affiliations:** ^1^ Department of Radiology State Key Laboratory of Cardiovascular Disease Fu Wai Hospital National Center for Cardiovascular Diseases Chinese Academy of Medical Sciences and Peking Union Medical College Beijing China; ^2^ Department of Radiology Fuwai Hospital Chinese Academy of Medical Sciences Shenzhen China; ^3^ Department of Radiology Bayannur Hospital Bayannur China; ^4^ Department of Radiology Baotou Central Hospital Baotou China; ^5^ Institute of cardiovascular disease the First Affiliated Hospital of Jinzhou Medical University Jinzhou China; ^6^ Department of Neurology Xuanwu Hospital Capital Medical University Beijing China

**Keywords:** angiogenesis, Atherosclerosis, lncRNA HCG11, miR‐26b‐5p, QKI‐5

## Abstract

Acute coronary syndrome caused by the rupture of atherosclerotic plaques is one of the primary causes of cerebrovascular and cardiovascular events. Neovascularization within the plaque is closely associated with its stability. Long non‐coding RNA (lncRNA) serves a crucial role in regulating vascular endothelial cells (VECs) proliferation and angiogenesis. In this study, we identified lncRNA HCG11, which is highly expressed in patients with vulnerable plaque compared with stable plaque. Then, functional experiments showed that HCG11 reversed high glucose‐induced vascular endothelial injury through increased cell proliferation and tube formation. Meanwhile, vascular‐related RNA‐binding protein QKI5 was greatly activated. Luciferase reporter assays and RNA‐binding protein immunoprecipitation (RIP) assays verified interaction between them. Interestingly, HCG11 can also positively regulated by QKI5. Bioinformatics analysis and luciferase reporter assays showed HCG11 can worked as a competing endogenous RNA by sponging miR‐26b‐5p, and QKI5 was speculated as the target of miR‐26b‐5p. Taken together, our findings revered that the feedback loop of lncRNA HCG11/miR‐26b‐5p/QKI‐5 played a vital role in the physiological function of HUVECs, and this also provide a potential target for therapeutic strategies of As.

## 1 INTRODUCTION

Vascular endothelial cells (VECs) injury and the blood vessel functional imbalance induce atherosclerosis plaque. The instability and rupture of vulnerable atherosclerotic plaque are the main reasons for cardiovascular and cerebral vascular events. The local neovascularization is closely associated with plaque stability,^1^, ^2^ because excessive angiogenesis is associated with intraplaque haemorrhage, which may contribute to plaque progression and rupture.^3^ Hyperglycaemia is known to impair endothelial cells (ECs) angiogenesis, such as in diabetic retinopathy (DR) or myocardial infarction (MI).^4^, ^5^, ^6^ Regarding the mechanism of angiogenesis, many angiogenic factors and signalling pathways have been shown to regulate blood vessel growth and morphogenesis.^7^ Recent studies have revealed important functions of lncRNAs in angiogenesis and cardio‐cerebrovascular disease.^8^

Long non‐coding RNAs (lncRNAs) are characterized by a length of more than 200 nucleotides and a lack of coding protein function. It is becoming widely accepted that several types of lncRNAs, which are involved in regulating the ECs proliferation, migration and angiogenic capacity, are considered to be the new markers for the diagnosis and prognosis of cardiovascular and cerebrovascular diseases.^9^, ^10^, ^11^ In atherosclerosis, as a molecular sponge for miR‐195, overexpression of lncRNA activated by tumour growth factor‐β (lncRNA‐ATB) increases ECs viability, migration and angiogenesis, along with up‐regulation of matrix metalloproteinase‐2 (MMP‐2), MMP‐9, and vascular endothelial growth factor (VEGF) that associated with stability of plaque.^12^ lncRNA TCONS_00024652 acts as a competitive endogenous RNA (ceRNA) that affects ECs proliferation and angiogenesis after TNF‐α stimulation by modulating miR‐21 expression.^13^ According to previous reports, lncRNA HCG11 was up‐regulated in atherosclerotic plaques, but the role of HCG11 in the progression of atherosclerotic plaque is unclear.^14^ Subsequently, Zhang Y et al showed that down‐regulation of HCG11 in prostate cancer (PCa) tissues was associated with poor survival of PCa patients.^15^ Xu Y et al found HCG11 increased cell viability, proliferation and migration ability in HepG2 cells via interaction with IGF2BP1, leading to activation of MAPK signalling, which eventually promoted the progression of HCC.^16^ As it is well known that the stability of advanced atherosclerotic plaque is closely related to intraplaque neovascularization, and in our present study, we found HCG11 was high expressed in vulnerable plaque and implemented preliminary research on the effect of angiopoiesis of HUVECs.

Mechanistically, in general, lncRNA exerts its biological effects by combining with competing endogenous RNAs to regulate target gene expression.^17^, ^18^ Bioinformatic evidence reveals the binding sites between HCG11 and miRNA‐26b‐5p. MiR‐26b‐5p is a member of the miR‐26 family. It has been confirmed as an important regulator in breast cancer and other pathological processes.^19^ MiR‐26b‐5p suppressed vascular mimicry (VM) and angiogenesis by down‐regulating the expression of VE‐cadherin, Snail and MMP2 and could inhibit the apoptosis of HCC cells ^20^; MiR‐26b‐5p inhibited liver fibrogenesis and angiogenesis through directly targeted PDGF receptor‐β. However, the role of miR‐26b‐5p in the progression of atherosclerosis is still unclear.

Using bioinformatics analysis approaches, we identified the RNA‐binding protein (RBP) quaking (QKI‐5) as a miR‐26b‐5p target mRNA in addition, catRAPID analysis predicted the binding potential of HCG11 and QKI‐5. QKI‐5 is a member of the RNA Signaling and Activation (STAR) family of RNA‐binding proteins. It plays an important regulatory role in accumulating ECs and to induce angiogenesis. Azam SH et al found that endothelial QKI‐5 expression remarkably correlated with angiogenic indices.^21^ Cochrane A et al found that the mouse ECs overexpressing QKI‐5 significantly improved angiogenesis and neovascularization and blood flow recovery in experimental hind limb ischaemia. The results highlight a clear functional benefit of QKI‐5 in neovascularization, blood flow recovery and angiogenesis.^22^

The present study was designed to explore the effect of HCG11 in the stability of atherosclerotic plaque. We measured the expression of HCG11 in human plasma samples. The previous study mentioned that high concentrations of glucose impaired angiogenesis.^5^ Our experiments found HG‐induced HUVECs reduced the expression of HCG11. HCG11 levels paralleled with QKI‐5, a key molecule involved in triggering angiogenesis. In addition to being able to combine directly with QKI‐5, we also found that HCG11 acts as sponge to regulate miR‐26b‐5p and further affects the expression of the target gene, QKI‐5. All results showed that HCG11 reversed HG‐induced HUVECs proliferation and angiogenesis inhibition via the miR‐26b‐5p/QKI‐5 feed back loop signalling pathway.

## 2 MATERIALS AND METHODS

### 2.1 Ethics statement

The clinical study was approved by the ethics committees of Inner Mongolia Medical University of China (No. YKD2015061). All procedures were in accordance with the Helsinki declaration. All patients gave their written informed consent to participate in the study. The data do not contain any information that could identify patients.

### 2.2 Clinical sample collection

The study population consisted of 290 patients with atherosclerosis vulnerable plaque, 178 stable plaque and 547 normal control people. The 1015 participants were consecutively recruited between April 2017 and May 2018 at Imaging Department of Baotou Central Hospital (Baotou, China). The patients underwent Integrated Imaging In Coronary Combined With Carotid And Cerebrovascular CT Angiography examinations. The exclusion criteria were as follows: (1) patients with history of contrast allergies, (2) patients with history of severe liver and renal insufficiency (glomerular filtration rate < 60ml/min), (3) valvular disease or other heart diseases such as cardiomyopathy or congenital heart disease (possible Affect the level of cellular inflammatory factors), (4) pregnant or lactating, lactating women. Collect all patients basic informations and follow‐up for 2 years. 200 cases of elbow venous blood samples were collected from patients and be processed immediately within 15 minutes. Each sample was frozen and stored at − 80℃. These samples were used for quantitative real‐time polymerase chain reaction (qRT‐PCR) analysis. Clinical characteristics of all patients are listed in Table 1.

**Table 1:** Baseline and characteristics of the patient population

	VP group (n = 290)	SP group (n = 178)	NG (n = 547)	*p* value
Clinical characteristics				
Gender, % (Male)	67.59 (196)	61.80 (110)	48.26 (264)	0.153
Age, years	58 ± 9	56 ± 15	52 ± 5	0.098
Mean Heart rate (bpm)	76.08 ± 12.66	77.36 ± 3.05	74.61 ± 13.84	0.516
Hypertension %(n)	47.2 (137)	44.9 (80)	59.2 (324)	0.001
Diabetes %(n)	26.20 (76)	24.71 (44)	3.47 (19)	0.001
BMI kg/m^2^	25.70 ± 3.58	25.03 ± 3.05	24.94 ± 3.72	0.034
Health habits				
Current cigarette user, % (n)	56.2 (163)	69.6 (124)	57.6 (315)	0.059
Current alcohol user, % (n)	33.8 (98)	33.7 (60)	34.9 (191)	0.108
Laboratory data				
FBG, mmol/L	7.47 ± 2.41	6.52 ± 2.03	6.03 ± 1.97	0.001
TG, mmol/L	2.48 ± 0.87	2.37 ± 0.82	1.86 ± 0.86	0.01
TC, mmol/L	4.34 ± 1.15	4.52 ± 1.21	4.50 ± 0.83	0.472
LDL‐C, mmol/L	2.40 ± 1.21	2.27 ± 1.07	2.13 ± 1.08	0.001
HDL‐C, mmol/L	1.02 ± 0.23	1.16 ± 0.25	1.25 ± 0.25	0.001
CRP, ug/L	13.200.256	11.89 ± 5.67	2.00 ± 1.13	0.001
CK, U/L	140.92 ± 98.39	97.55 ± 46.88	98.33 ± 13.53	0.013
CK‐MB, U/L	1.73B,13.	2.43B,13.	2.58B,13.	0.420

Note

Table demonstrating baseline and characteristics of the patient population.

*Abbreviations*: CK, creatine kinase; CK‐MB, creatine kinase‐MB; CRP, C‐reactive protein; FBG, fasting blood glucose; HDL‐C, high‐density lipoprotein cholesterol; LDL‐C, low‐density lipoprotein cholesterol; NG, normal group; SP, stable plaque; TC, total cholesterol; TG, triglyceride; VP, vulnerable plaque.

### 2.3 Definition of CT plaque characteristics

There are four points for diagnosing vulnerable atherosclerotic plaques: (1) Low attenuation plaque (LAP): average density ≤ 30 HU from 3 random region‐of‐interest measurements, with approximately 0.5 to 1.0 mm2 in non‐calcified low CT attenuation portion of the plaque; (2) Positive remodelling (PR): remodelling index ≥ 1.1; (3) Spotted calcification (SC): average density> 130 HU, diameter < 3 mm in any direction, length of the calcium < 1.5 × the vessel diameter, and width of the calcification less than two‐thirds of the vessel diameter and; (4) "Napkin ring" sign: ring‐like attenuation pattern with peripheral high attenuation tissue surrounding a central lower attenuation portion. According to CT characteristics, patients were three groups: vulnerable plaque (VP) group, stable plaque (SP) group and normal group (NG).

### 2.4 Follow‐up and end‐points

After integrated coronary‐carotid‐cerebral computed tomography angiography (ICCC‐CTA), the three groups of patients received telephone follow‐up. The follow‐up time was as follows: 4, 8, 12, 16, 20 and 24 months. Defined end‐points were angina pectoris, MI or stroke, stents or bypass grafts and sudden death. Retrospective analysis of the incidence of MACCE in patients in the VP group and SP group, at different onset periods, were performed to validate the predictive model of plaque vulnerability, using ICCC‐CTA.

### 2.5 Cell culture and treatment conditions

Human umbilical vein endothelial cells (HUVECs) were purchased from American Type Culture Collection (ATCC). And the cells were cultured in M199 medium containing 10% foetal bovine serum and the cultivation environment was humidified atmosphere with 5% CO_2_ at 37°C. HUVECs were seeded at 3x10^5^cells/well in 6‐well plates and treated with conditional concentrations (5.5, 11 or 33 mM for 24 h) or for different points (33 mM for 0, 12, 24 or 48 h) with glucose.

### 2.6 Fluorescence in situ hybridization (FISH)

The *HCG11* FISH probes were designed and synthesized by RiboBio (Guangzhou, China), according to the manufacturer's instructions from fluorescent In Situ Hybridization Kit purchased from RiboBio (Guangzhou, China), firstly, the probes were combined with dye, and then HUVECs were fixed with 4% PFA, perforated with 0.5% Triton X‐100, after treatment with protease K reagent, the cells were incubated with pre‐hybridization solution at 55℃ for 1h, after remover the pre‐hybridization solution, the cells were hybridized with hybridization solution containing probes (1ontal) at 55℃) for 16‐20h, and then the cells were incorporated with DAPI for 5 min, after twice washed with pre‐hybridization solution, the probe signals were detected under a fluorescence microscope (Leica, Germany).

### 2.7 Cell transfection

For the transfection, lipofectamine 2000 was used to transfect miR‐26b‐5p mimics, miR‐26b‐5p inhibitor, overexpression of HCG11, knock down of QKI‐5 (sh‐QKI‐5‐Sense: 5’‐ccggAAGCACCTACAGAGATGCCAACTCGAGTTGGCATCTCTGTAGGTGCTTTTTTTg‐3’; sh‐QKI‐5‐Antisense:5’‐aattcAAAAAAAGCACCTACAGAGATGCCAACTCGAGTTGGCATCTCTGTAGGTGCTT‐3’) and corresponding negative controls. Briefly, after seeding 1 × 10^6^ cells into 6‐well plate, all transfections were mixed with lipofectamine 2000 (Invitrogen, USA) in FBS free Opti‐MEN Medium (Thermo Fisher), and the final concentration of plasmids was 2.5 μg/well, and miR‐26b‐5p mimics or inhibitors were transfected with 40 nmol/ml. The transfection mixtures were then added to cells and incubated for 6 hours. After that, the medium was replaced by fresh normal growth medium, and the transfection efficiency was validated with qRT‐PCR or Western blot.

### 2.8 EdU proliferation assay

For the cell proliferation assay, HUVECs were seeded in 96‐well plate. After transfection of the cells as described above, the cells were treated with conditional concentrations (5.5, 11 and 33 mM)or time points(0, 12, 24 and 48 h) of glucose, the proliferation was detected through the EdU Cell Proliferation Assay Kit (Ribobio, china). According to the manufacturer's instructions, firstly, the cells were incubated with EdU for 4 h, following fixation, permeabilization and Edu staining, and last, the cells were stained with DAPI for 5 min, the percentage of the cells incorporated EdU was assayed using a fluorescence microscope.

### 2.9 Matrigel‐based tube formation assay

The capability of HUVECs to form capillary tube‐like structures was assessed by the Matrigel‐based tube formation assay. 50 μl of Matrigel was added to a pre‐cooling 96‐well plate and solidified at 37°C for 1 hour. After that, the pre‐transfected HUVECs were harvested and cultured on the Matrigel‐coated plate for another 8 hours, with or without glucose treatment (33mM). The tube formation effects were observed, and representative images were captured using a light microscope (magnification, ×400).

### 2.10 Real‐time fluorescent quantitative PCR (qRT‐PCR)

After a series of above‐mentioned processing, the total RNA was extracted from HUVECs using RNA isolation kit (Invitrogen, USA) according to the manufacturer's instructions. Next, RNA was reversed transcript into complementary DNA (cDNA) by PrimeScript^TM^ RT reagent kit (Perfect Real Time) (TaKaRa, Japan). The synthesized cDNA was used to perform PCR on ABI 7500 fast Real‐Time PCR System. The sequences of the primers were as follows: HCG11, Forward, 5’‐GCTCTATGCCATCCTGCTT‐3’ and Reverse, 5’‐TCCCATCTCCATCAACCC‐3’. QKI‐5, Forward, 5’‐CTGTCATGCCAAACGGAAC‐3’andReverse, 5’‐GATGGACACGCATATCGTG‐3’. GAPDH, Forward, 5’‐AGGTCGGTGTGAACGGATTTG‐3’ and Reverse, 5’‐TGTAGACCATGTAGTTGAGGTCA‐3’. For miRNA expression assay, cDNA of miR‐26b‐5p was synthesized by All‐in‐one miRNA qRT‐PCR Reagent Kits (GeneCopoeia, Rockville, MD, USA). The primers of U6 and miR‐26b‐5p were constructed by GeneCopoeia. U6 and GAPDH were used as housekeeping gene. Relative levels of gene expression were calculated via the 2^‐ΔΔCt^ method.

### 2.11 Western blot and antibodies

HUVECs were cultured and transfected as described in Cell Culture and Transfection. After transfection, cells were treated with glucose (33 mg/ml) for another 24 hours. After the treatment, the cells were collected and washed twice with phosphate‐buffered saline. The total proteins were extracted by adding 50 μl radio immunoprecipitation assay lysis (with 1% phenylmethylsulphonyl fluoride and 10 mM NaF) into the cells for 30 minutes. For the Western blot assay, the total proteins were separated by SDS‐PAGE and transferred to polyvinylidene fluoride membranes. After that, the membranes were blocked with 4% non‐fat milk for 1 hour and then incubated with diluted QKI‐5 (#AB9904, Millipore Company, USA) a primary antibody overnight at 4°C. The membranes were afterwards washed and incubated with 1:10,000 dilution of the second antibody for 1 hour and were detected with the enhanced chemiluminescence system. Relative intensities were analysed by Quantity OneR software (Bio‐Rad, Hercules, CA).

### 2.12 Bioinformatics analysis and luciferase assay

To explore the regulatory mechanism of lncRNA HCG11 in AS, RNA‐RNA and protein‐RNA interaction networks were analysed using StarBase v2.0 (http://starbase.sysu.edu.cn/) and (http://service.tartaglialab.com/page/catrapid_group ). In addition, we used catRAPID to predict the binding rate of HCG11 and QKI to verify their binding potential.

The interaction between miR‐26b‐5p and HCG11 or QKI 3’UTR was verified by dual‐luciferase reporter gene assay. The luciferase reporter plasmids were constructed by Ribobio (guangzhou, china), which containing wild‐type HCG11, mutated HCG11 and wild‐type QKI or QKI mutated at the putative miR‐26b‐5p binding sites. These vectors were transfected into HUVECs with either miR‐26b‐5p mimics or a negative control with transfection reagent (Thermo Fisher Scientific, CA, USA). After 48 h of transfection, the measurement of luciferase activity was determined by Dual Luciferase Assay System (Promega, WI, USA) according to the manufacturer's instructions.

### 2.13 RNA‐binding protein immunoprecipitation (RIP) assay

For RIP experiments, we used a Research Science RIP assay kit (R&S Biotechnology, Pudong, Shanghai, China). Briefly, cells were collected and lysed incomplete RIP lysis buffer. After centrifugation, the supernatants were precleared with protein A beads (Roche, Mannheim, Germany) in lysis buffer, then the lysates were conjugated to anti‐QKI‐5 antibody (Abcam, Shanghai, China) and IgG antibodies for 4 h at 4℃. Samples were incubated with proteinase K (20 mg/μl) master mix with shaking to digest the proteins and the immunoprecipitated RNA was isolated. HCG11 level in the precipitates was determined by qRT‐PCR analysis.

### 2.14 Statistical analysis

All data were statistically analysed using the Statistical Package for the Social Sciences (SPSS) version 20.0 software (SPSS Inc, Chicago, IL, USA). All experiments were repeated 3 times independently, and the data were expressed as mean ± SD. Comparison of paired design between the two groups with positive distribution and homogeneity of variance was analysed by paired t test, otherwise non‐paired t test was applied. Statistical analysis among more than two groups was conducted by one‐way analysis of variance (ANOVA) or two‐way ANOVA followed by a Tukey post hoc test. Correlations between HCG11 and miR‐26b‐5p, HCG11 and LDL or FBG were analysed using the Spearman's correlation test. Statistical significance is shown as described in the figure legends. *P* < .05 indicates the difference was statistically significant.

## 3 RESULTS

### 3.1 Association of plaque characteristics with MACCE and clinical analysis

**Figure 1:**
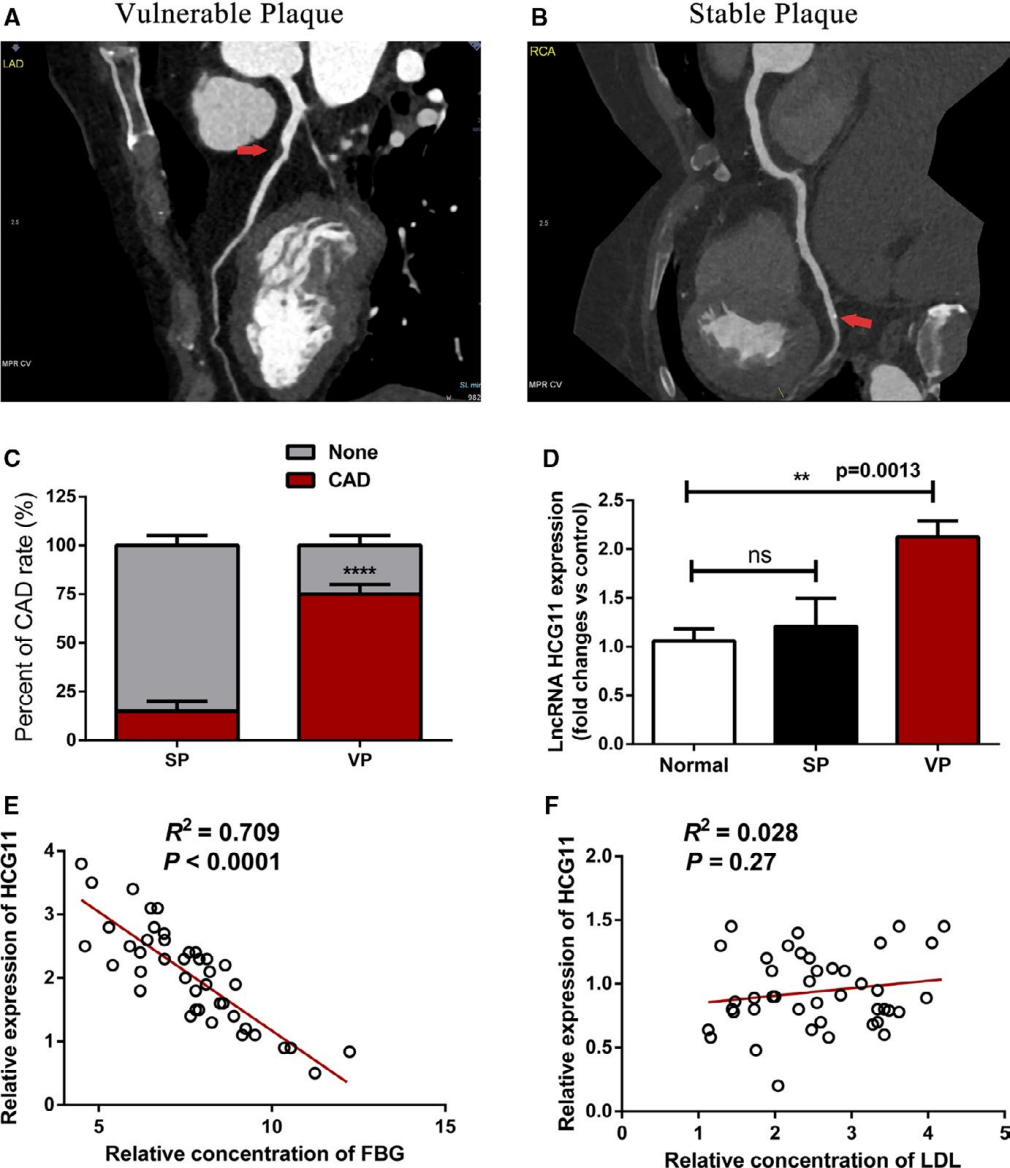
Identification elevated expression of HCG11 in vulnerable plaque and is negatively correlated with FBG. (A and B) Initial analysis for atherosclerotic plaque types using CTA, red arrow indicates the location of plaque, panel A presents vulnerable plaque and panel B presents stable plaque. (C) Cardiovascular event rate of patients with vulnerable plaque and stable plaque. (D) The expression of HCG11 in normal and patients with vulnerable plaque or stable plaque was evaluated by qRT‐PCR, normal is used as control, data represent the mean ± SD, **,*P* < .01. (E) Identification relationships of HCG11 and fasting blood glucose in 43 patients with vulnerable plaque, *R* ^2^ = 0.709, *P* < .0001,(F) Verification relationships of HCG11 and low‐density lipoprotein in 43 patients with vulnerable plaque, *R* ^2^ = 0.028, *P = *.27

Firstly, to determine if the plaques in the atherosclerotic patients were stable or vulnerable, all patients were scanned with SIEMENS dual source (DS)—integrated Imaging In Coronary Combined With Carotid And Cerebro vascular CT Angiography. Typical patients with vulnerable plaque and stable plaque by ICCC‐CTA were presented in Figure 1A‐B. Based on the patient follow‐up, 149 patients were lost, 758 patients had no events, and 108 patients had MACCE. The rate of MACCE was 23.07%, this included 81 patients with vulnerable atherosclerotic plaques and 27 patients with stable atherosclerotic plaques. The proportion of different MACCE between VP and SP groups is shown in supplementary Figure 1A. We constructed a graph of incidence based on the time that patients had MACCE (supplementary Figure 1B). Our analysis found that patients with vulnerable plaques are more likely to have coronary heart disease (CAD) (Figure 1C). The baseline and clinical characteristics of the study populations are shown in Table 1. Comparison of some parameters including Hypertension, FBG, TG, HDL and LDL among the three groups showed statistically significant differences.

### 3.2 LncRNA HCG11 is elevated expressed in vulnerable plaque and negatively correlated with FBG

Here, we investigated the lncRNA HCG11 expression pattern in plasma of patients in different group. Quantitative RT‐PCR (qRT‐PCR) showed that lncRNA HCG11 level was significantly higher in vulnerable plaque group, compared with that of stable plaque group (*p*＜0.05).While there was no significantly difference in stable plaque group, compared with their corresponding controls (Figure 1D). Using Spearman's correlation test, we analysed the negative correlation between the lncRNA HCG11 expression and the FBG expression level in patients of vulnerable plaque (Figure 1E), while there is no correlation between lncRNA HCG11 and LDL (Figure 1F). Together, these findings indicated the potential involvement of lncRNA HCG11 in stability of plaque.

### 3.3 High glucose inhibited proliferation and tube formation of HUVECs, simultaneously reduced lncRNA HCG11 expression

**Figure 2:**
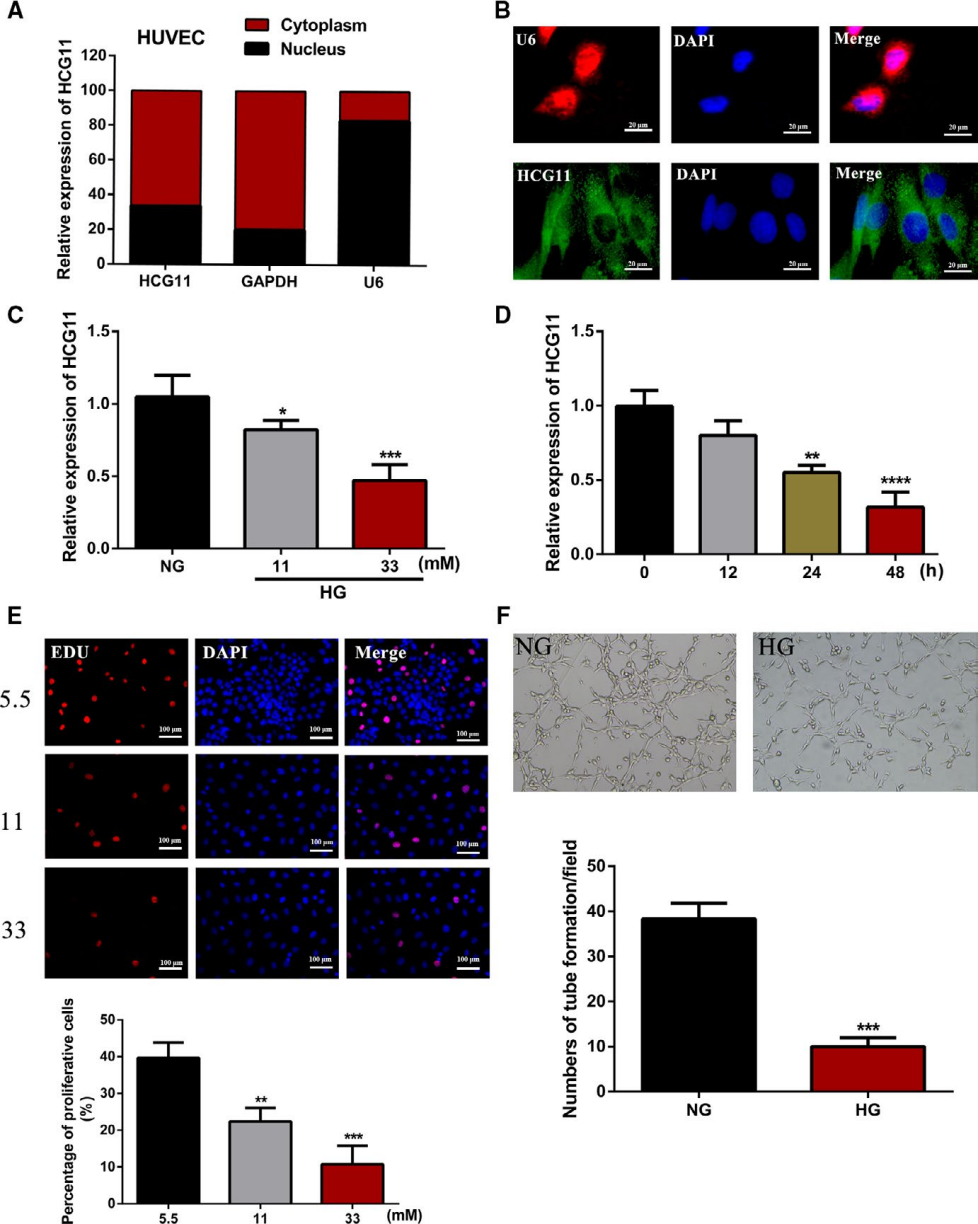
The proliferation, tube formation ability and expression of lncRNA HCG11 in different concentration of glucose. (A) The cellular localization of lncRNA HCG11 in HUVEC was identified by qRT‐PCR after separation of the nuclear and cytoplasmic compartments, GAPDH was used as a cytoplasmic transcript and U6 is a nuclear RNA. (B) RNA FISH indicated that lncRNA HCG11 is localized mainly in the cytoplasm of HUVECs (Scale bar, 20 μm). (C)The expression of lncRNA HCG11 was determinated in the presence of NG, or HG (11, 33 mM) by qRT‐PCR, NG is a control group, **, *P* < .01, ****, *P* < .0001. (D) The expression of lncRNA HCG11 was determined in different points of time with 33 mM concentration of glucose, 0 group is used as control, **, *P* < .01, ***, *P* < .001.(E) The proliferation capacity of HUVECs in different concentrations of glucose in 24h was measured by the EdU staining(200X), the lower panel showed the statistical data and described as mean ± SD, *, *P* < .05, ****, *P* < .0001. (F) The tube formation of HUVECs in NG and 33 mM conditions

To investigate the role of LncRNA HCG11 in the progression of HUVECs, firstly, based on the RNA FISH and subcellular fractionation assay, LncRNA HCG11 was enriched in the cytoplasm of HUVECs (Figure 2A‐B). The above data indicated that LncRNA HCG11 is negative correction with FBG in patients of vulnerable plaque, and then, the expression level of LncRNA HCG11 was detected in different concentrations of glucose in vitro, qRT‐PCR showed that LncRNA HCG11 is down‐regulated in high glucose, and the more glucose concentrations resulted in the lower expression of LncRNA HCG11 (Figure 2C), HCG11 also regulated by different time points at 33 mM condition (Figure 2D). These data indicated that high glucose could lead to an obvious decrease of LncRNA HCG11 expression in a time‐dependant and dose‐dependant manner. Subsequently, the EdU assay and Matrigel‐based tube formation assays were performed to detect the role of HG in cell proliferation and tube formation. The results showed that the proliferation and tube formation of HUVECs were profoundly decreased after incubated with different level of HG (Figure 2E‐F). These finding indicated that HG‐induced inhibition of proliferation and tube formation may corrected with LncRNA HCG11.

### 3.4 Overexpression of lncRNA HCG11 reversed HG‐induced inhibition of proliferation and tube formation of HUVECs

**Figure 3:**
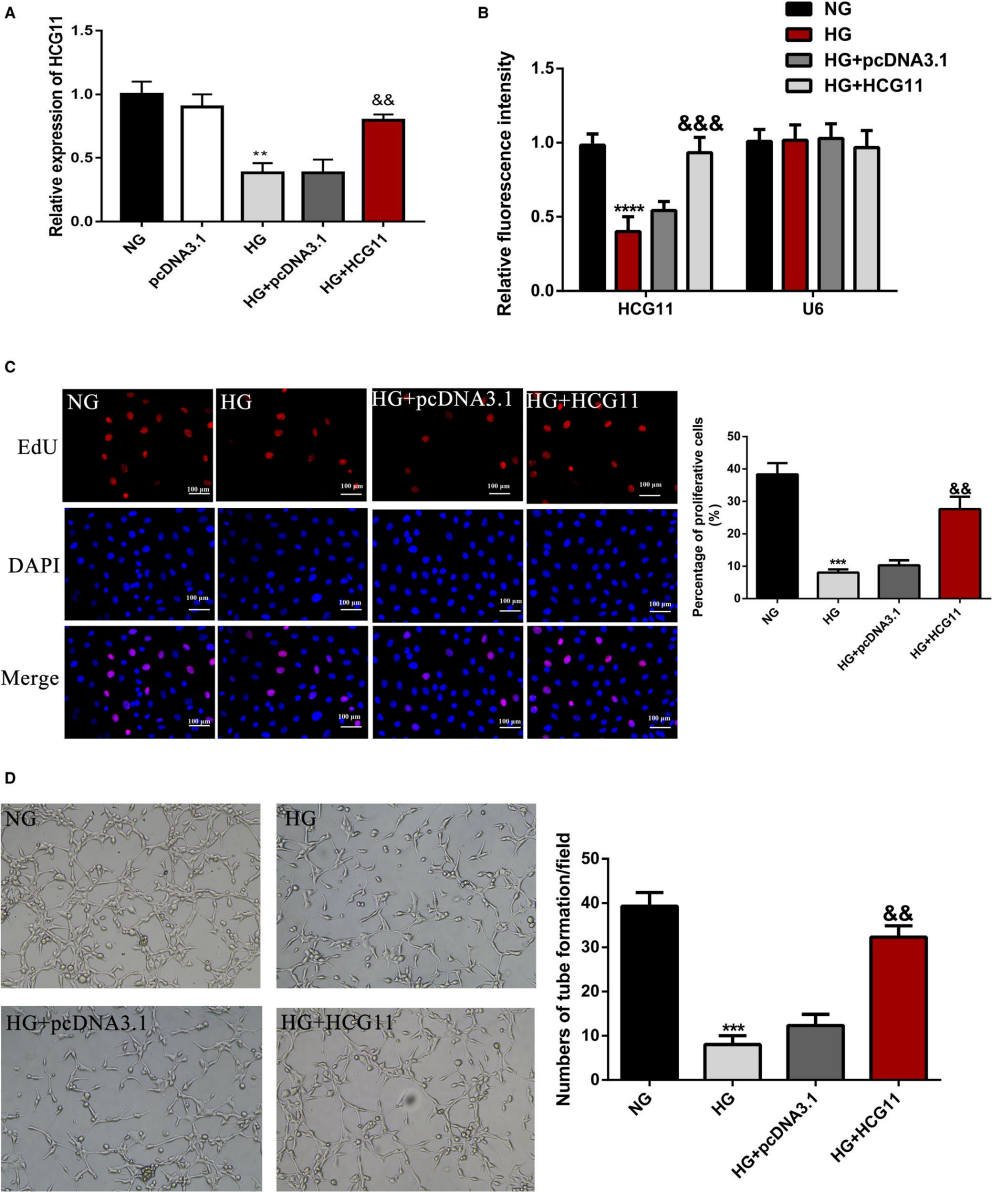
Overexpression of lncRNA HCG11 reversed HG‐induced inhibition of proliferation and tube formation of HUVECs. HUVECs were treated with NG, pcDNA3.1 or lncRNA HCG11 in the presence of HG or HG alone. (A) The expression of lncRNA HCG11 of HUVECs in different treatments by qRT‐PCR, ^**^ *P* < .01 vs NG group, ^&&^ *P* < .01 vs HG + pcDNA3.1 group. (B) Statistic analysis of fluorescence intensity of lncRNA HCG11 FISH staining, U6 is a negative control. (C) HCG11 reversed the proliferation of HUVECs in HG conditions, the left panel is EdU staining and the right panel analysis the percentage of positive cells, ^***^ *P* < .001 vs NG group, ^&&^ *P* < .01 vs HG + pcDNA3.1 group. (D) Matrigel tube formation assay was used to evaluated overexpression of lncRNA HCG11 in the presence of HG on the tube formation of HUVECs.^***^ *P* < .001 vs NG group, ^&&^ *P* < .01 vs HG + pcDNA3.1 group

To explore the function of lncRNA HCG11 in the proliferation and tube formation in HG treatment, we constructed lncRNA HCG11 overexpression vectors in HUVECs, lncRNA HCG11 is overexpressed after transfected with pcDNA/HCG11, the pcDNA3.1 was regarded as a negative control, qRT‐PCR and RNA FISH assay were used to examine the overexpression efficiency (Figure 3A‐B). We performed proliferation assay and tube formation assays to gain insight into the role of lncRNA HCG11 in HG‐induced cell proliferation and tube formation inhibition. As shown in (Figure 3E), EdU proliferation assay found that 24h treatment with glucose (33 mM) inhibited the growth of HUVECs significantly, and overexpressed of lncRNA HCG11 reversed the cell growth inhibition induced by HG. According to the tube formation assay, we founded the similar effects of lncRNA HCG11 (Figure 3F), these findings suggested that lncRNA HCG11 participated in HG‐induced proliferation and tube formation inhibition of HUVECs.

### 3.5 QKI‐5 is a target gene of HCG11 and is involved in lncRNA HCG11 mediated HUVECs proliferation and angiogenesis

**Figure 4:**
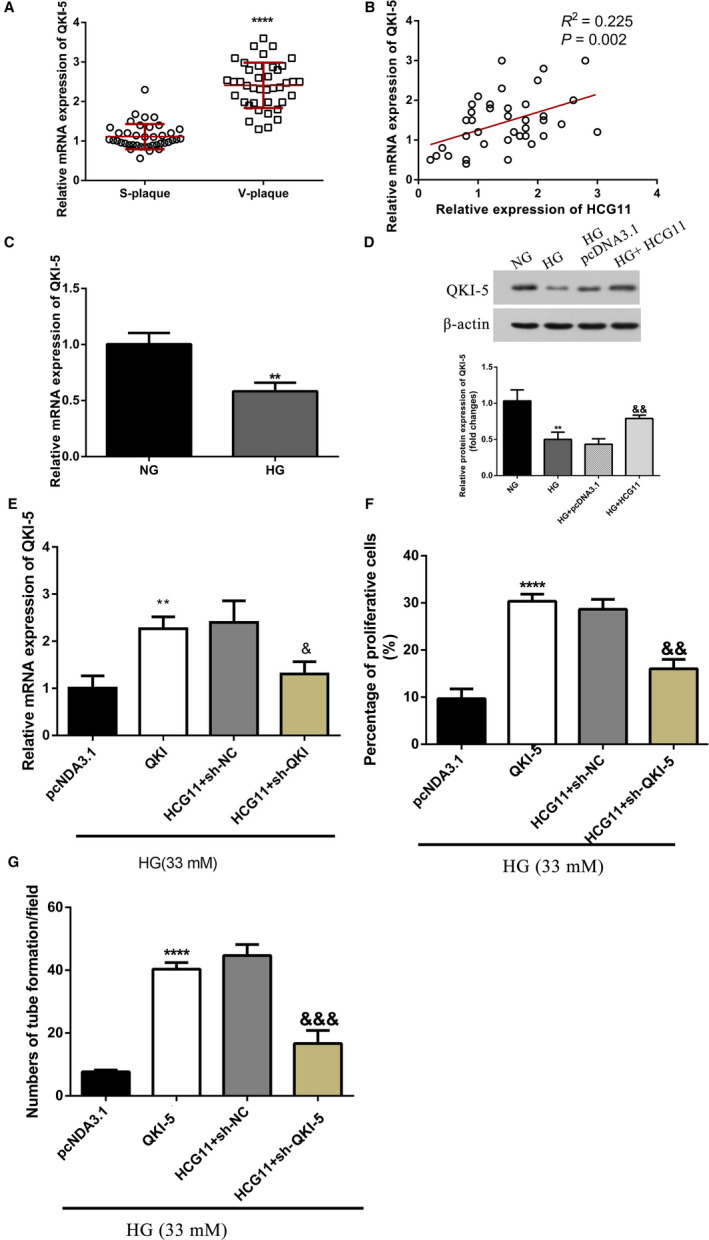
QKI‐5 is a downstream target of lncRNA HCG11, down‐regulation of QKI‐5 suppressed HCG11 mediated proliferation and tube formation of HUVECs. (A) The expression of QKI‐5 mRNA in plasma of patients in different atherosclerotic plaque group. Data are presented as the mean ± SD (n = 43), ^****^ *P* < 0.0001vs S‐plaque group. (B)Identification relationships of lncRNA HCG11 and QKI‐5 in 43 patients with vulnerable plaque by pearson's correlation analysis, *R* ^2^ = 0.225, *P* = .002. (C) The expression of QKI‐5 treated with NG or HG (33mM) by qRT‐PCR, ^**^ *P* < .01 vs NG group. (D) Overexpression of lncRNA HCG11 increased the protein level of QKI‐5 in HG condition, ^**^ *P* < .01 vs NG group, ^&&^ *P* < .01 vs HG + pcDNA3.1 group. (E) The expression of QKI‐5 of HUVECs in different treatments by qRT‐PCR, ^**^ *P* < .01 vs pcDNA 3.1 group, ^&^ *P* < .05 vs HCG11 + sh‐NC group. (F) The co‐effects of lncRNA HCG11 and QKI‐5 in the proliferation of HUVECs was evaluated by EdU assay, ^****^ *P* < .0001 vs pcDNA 3.1 group, ^&&^ *P* < .01 vs HCG11 + sh‐NC group. (G) The co‐effects of lncRNA HCG11 and QKI‐5 in tube formation was detected by matrigel tube formation assay,^**^ *P* < .01 vs pcDNA 3.1 group, ^&&^ *P* < .01 vs HCG11 + sh‐NC group

Previous studies have demonstrated that QKI‐5 is a key molecule involved in triggering angiogenesis^23^ and is dysregulated in many cancers.^24^ Quantitative RT‐PCR showed that the level of QKI‐5 mRNA was significantly higher in vulnerable plaque group, compared with that in stable plaque group (*p*＜0.0001) (Figure 4A). In addition, correlation analyses showed that QKI‐5 expression was positively associated with LncRNA HCG11 in plasma of patients with vulnerable plaques (*p*＜0.05) (Figure 4B). In vitro experiment also showed that QKI‐5 was down‐regulated in HG‐treated HUVECs (Figure 4C). To verify whether the angiogenesis effects of lncRNA HCG11 are mediated by QKI‐5, we further detected the expression of QKI‐5 with lncRNA HCG11 overexpression in HG condition. Western Blot Analysis indicated that QKI‐5 protein levels were increased after overexpression lncRNA HCG11(Figure 4D). Moreover, qRT‐PCR was used to examine the transfection efficiency of QKI‐5 (Figure 4E), EdU and tube formation assay showed that knockdown of QKI‐5 suppressed lncRNA HCG11‐induced HUVECs proliferation and tube formation (Figure 4F‐G). These results indicate that lncRNA HCG11 might play a role in proliferation and angiogenesis through QKI‐5.

### 3.6 LncRNA HCG11 directly interacted with QKI‐5 in HUVECs

**Figure 5:**
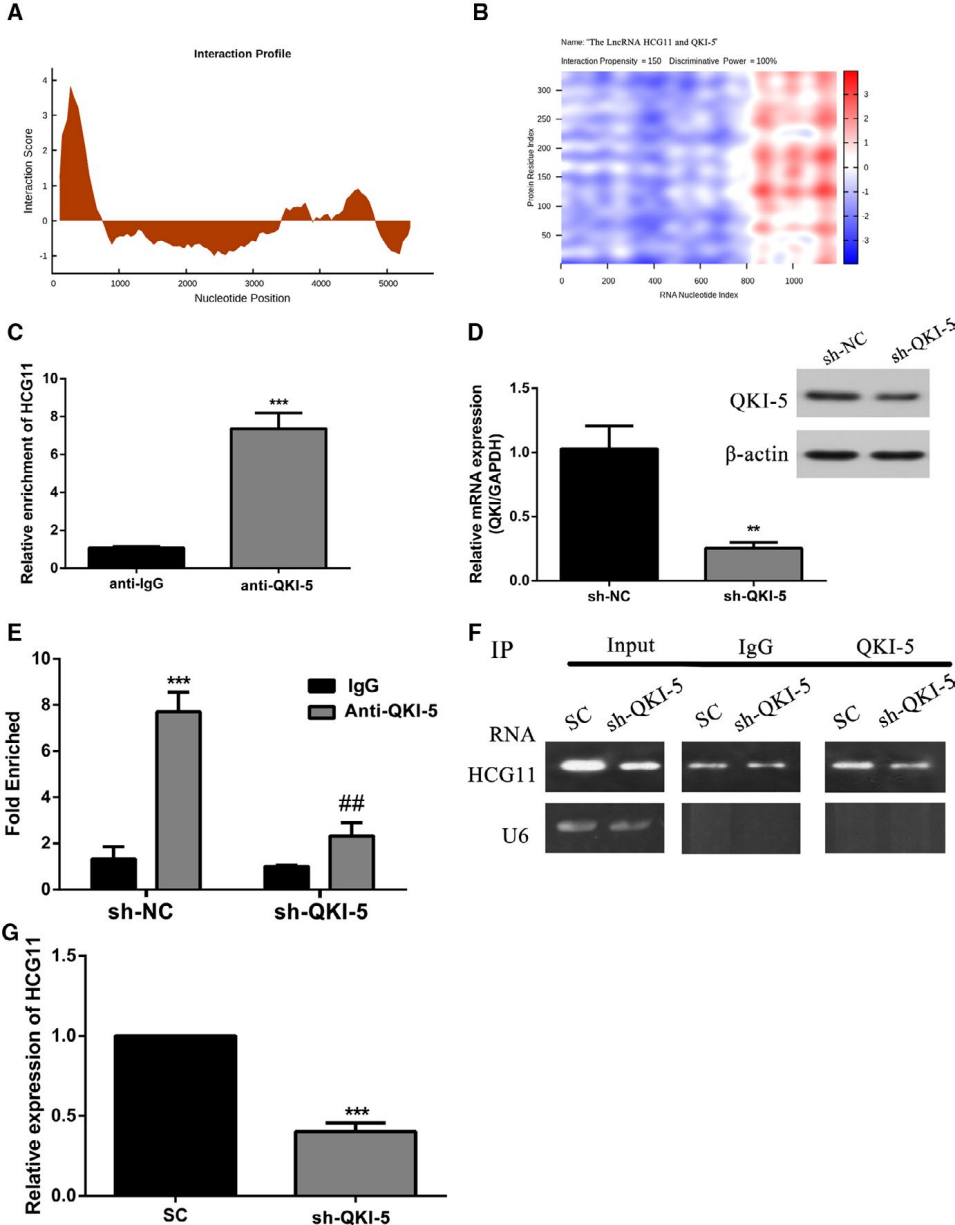
QKI‐5 interacted with lncRNA HCG11 and regulate lncRNA HCG11 expression. (A‐B) Prediction the interaction between lncRNA HCG11 and QKI‐5. The interaction profile represents the interaction score (y‐axis) of the protein along the RNA sequence (x‐axis), giving information about the transcript regions that are most likely to be bound by QKI‐5. The actual prediction result of lncRNA HCG11 and QKI‐5 was shown as a heat‐map, the colour presented different interaction score of the individual amino acid and nucleotide pairs. The *cat*RAPID identified the interaction between lncRNA HCG11 and QKI‐5 with confidence (Interaction Propensity = 150, Discriminative Power = 100%). (C) The enrichment of lncRNA HCG11 in anti‐IgG and anti‐QKI‐5 was detected by RNA‐IP assay, ^***^ *P* < .001 vs anti‐IgG group. (D) The mRNA and protein expression level of QKI‐5, ^**^ *P* < .01 vs sh‐NC group. (E) Cellular extracts of HUVECs transfected with sh‐NC or sh‐QKI‐5 were immunoprecipitated with IgG or QKI‐5 antibodies, and the target lncRNA in the immunoprecipitation material was detected by quantitative RT‐PCR (i) and semi‐quantitative RT‐PCR (ii) using primers against lncRNA HCG11. U6 was analysed is a negative control, ^***^ *P* < .001,^**^ *P* < .01 vs anti‐IgG group

As a RNA‐binding protein, QKI‐5 favoured interacted with RNAs to perform its function. To verified potential interaction between lncRNA HCG11 and QKI‐5, catRAPID was used to rapidly evaluate the interaction tendency of lncRNA HCG11 and QKI‐5 based on the secondary structure, hydrogen bonding and molecular interatomic forces. The prediction revealed that there existed an interaction between lncRNA HCG11 and QKI‐5 with a IP value of 150 and DP value of 100% (Figure 5A‐B). Then, to further verified this prediction, RIP experiment was applied to detected whether they can interact with each other, the relative enrichment levels of lncRNA HCG11 was increased in the anti‐QKI‐5 group than the anti‐IgG group (Figure 5C). Our previous data showed QKI‐5 is a down regulator of lncRNA HCG11, weather QKI‐5 played a role in the regulation of lncRNA HCG11, this deserves further research. Knockdown of QKI‐5 rapidly reduced the enrichment of lncRNA HCG11 with anti‐QKI‐5 (Figure 5D‐F), and the total expression level of lncRNA HCG11 in HUVECs also reduced (Figure 5G). These results indicated QKI‐5 promotes lncRNA HCG11 stability through directly interacted with LncRNA HCG11.

### 3.7 LncRNA HCG11 functions as miR‐26b‐5p sponge in HG‐induced HUVECs

**Figure 6:**
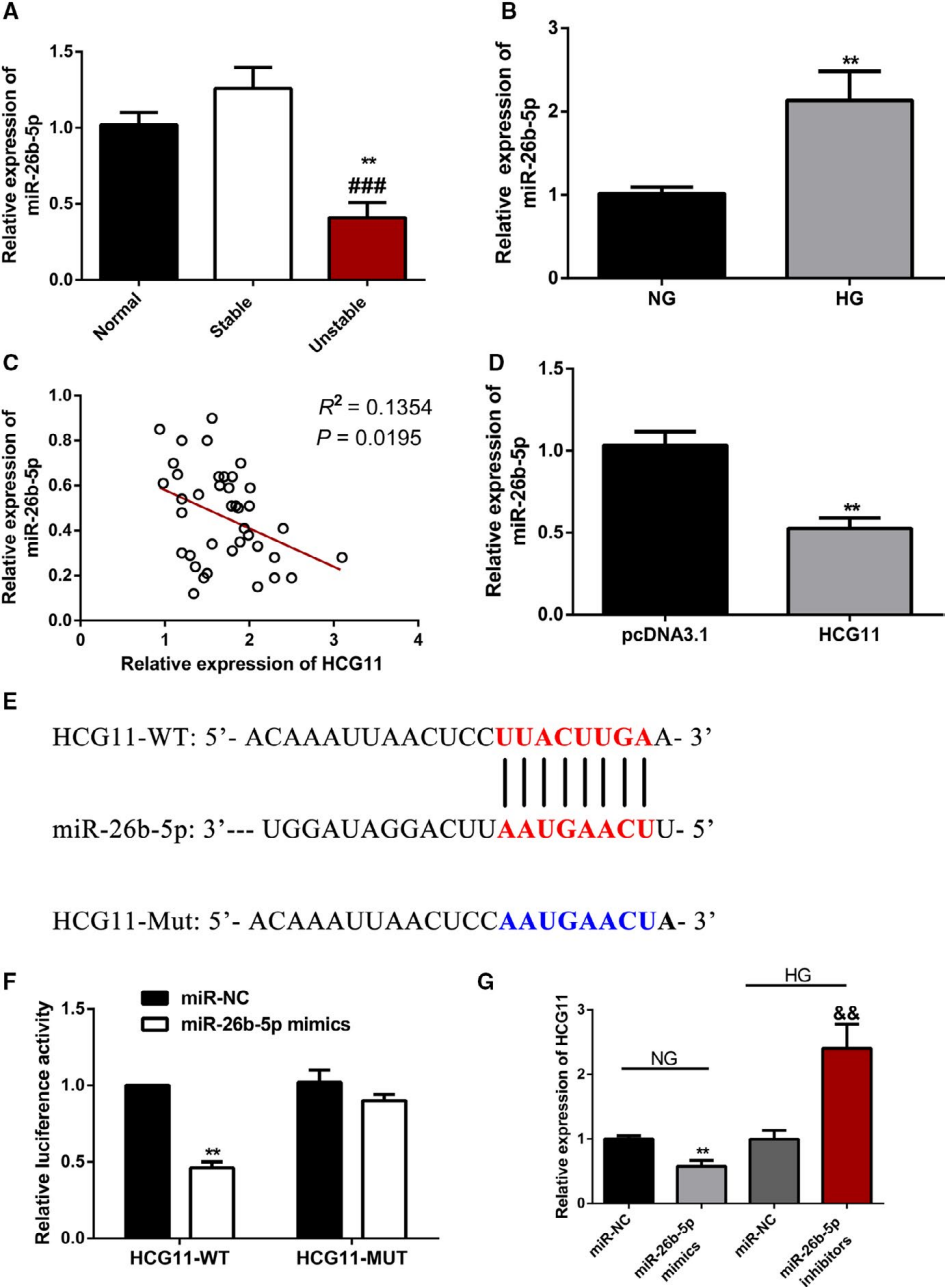
LncRNA HCG11 functions as miR‐26b‐5p sponge in HG‐induced HUVECs. (A) The expression of miR‐26b‐5p in plasma of patients in different atherosclerotic plaque group. Data are presented as the mean ± S.D, ^**^ *P* < .01 vs normal group, ^###^ *P* < .001 vs sp group. (B) Effects of high glucose on the expression of miR‐26b‐5p level in HUVECs,^**^ *P* < .01 vs NG group. (C) The correlation between the lncRNA HCG11 and miR‐26b‐5p, *R* ^2^ = 0.135, *P* = .0195. (D) Effect of HCG11 overexpression on miR‐26b‐5p expression level, ^**^ *P* < .01 vs pcDNA3.1 group. (E) Schematic representation of the putative miR‐26b‐5p binding sites in lncRNA HCG11. (F) the luciferase reporter assay was used to examine the relative luciferase activity in HUVECs cotransfected with lncRNA HCG11‐WT or lncRNA HCG11‐Mut reporter plasmid together with miR‐26b‐5p mimics, ^**^ *P* < .01 vs miR‐NC group. (G) The expression of lncRNA HCG11 in the present with miR‐26b‐5p mimics or inhibitors together with NG or HG condition, ^**^ *P* < .01,^&&^ *P* < .01 vs miR‐NC

**Figure 7:**
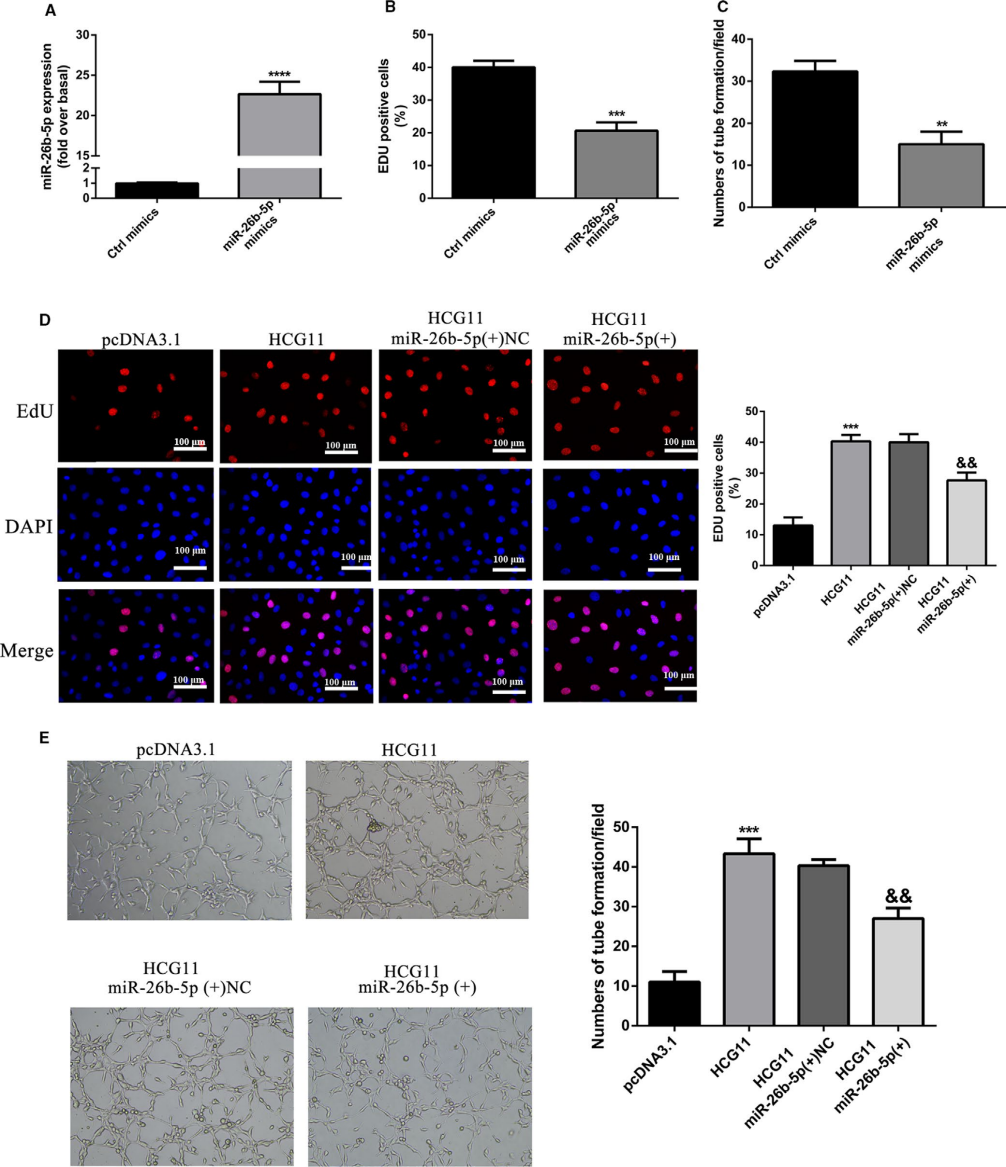
MiR‐26b‐5p functionally mediated lncRNA HCG11 induced proliferation and tube formation. (A‐C) Increased expression of miR‐26b‐5p promoted proliferation and tube formation of HUVECs,^**^ *P* < .01, ^***^ *P* < .001, ^****^ *P* < .0001 vs Ctrl mimics group. (D) miR‐26b‐5p mimics impaired lncRNA HCG11 induced proliferation of HUVECs in HG treatment, ^***^ *P* < .001 vs pcDNA3.1 group, ^&&^ *P* < .01 vs lncRNA HCG11 + miR‐26b‐5p(+)NC group. (E) The co‐effects of lncRNA HCG11 and miR‐26b‐5p on the tube formation of HUVECs was determinated by matrigel tube formation assay, ^***^ *P* < .001 vs pcDNA3.1 group, ^&&^ *P* < .01 vs lncRNA HCG11 + miR‐26b‐5p(+)NC group, magnification (100X)

In order to research the mechanism of lncRNA HCG11 regulated QKI‐5, Starbase prediction software showed miR‐26b‐5p is a competitive miRNA between lnRNA HCG11 and QKI‐5. So we assessed whether lncRNA HCG11 acts as a competitive endogenous RNA to miR‐26b‐5p and inhibits the expression of miR‐26b‐5p. Quantitative RT‐PCR (qRT‐PCR) shows that miR‐26b‐5p level was significantly lower in vulnerable plaque group compared with stable plaque group (*p*＜0.01). And it was no significantly difference between stable plaque group and normal controls (Figure 6A). In vitro data showed treatment with glucose (33mM) for 24h up‐regulated the expression of miR‐26b‐5p (Figure 6B). Correlation analyses showed that lncRNA HCG11 expression was negatively associated with miR‐26b‐5p level in plasma of patients with atherosclerotic vulnerable plaques (Figure 6C). Overexpression of lncRNA HCG11 significantly down‐regulated the level of miR‐26b‐5p (Figure 6D). To verify whether the angiogenesis effects of lncRNA HCG11 are completely mediated by miR‐26b‐5p, we next conducted dual‐luciferase reporter assays. The results demonstrated that cotransfection with wild‐type lncRNA HCG11 (HCG11‐WT) plasmids and miR‐26b‐5p mimics remarkably reduced the luciferase activities in HUVECs, whereas the luciferase activities were not changed when the cells were cotransfected with mutant lncRNA HCG11 (LUCAT1‐MUT) plasmids as well as miR‐26b‐5p mimics (Figure 6E‐F). Moreover, the relative expression of lncRNA HCG11 was significantly decreased in HG‐induced HUVECs when they were transfected with miR‐26b‐5p mimics, whereas silence of miR‐26b‐5p mimics resulted in notably decreased expression of lncRNA HCG11 in HG‐induced HUVECs (Figure 6G). Increased expression of miR‐26b‐5 reduced the endothelial cells proliferation and tube formation (Figure 7A‐C). Further research found miR‐26b‐5p mimics abrogated the effects of lncRNA HCG11 on high glucose‐induced angiogenesis inhibition (Figure 7D‐E). All results suggested that miR‐26b‐5p was a target of lncRNA HCG11 and interacts with HCG11 to promote angiogenesis.

### 3.8 LncRNA HCG11/MiR‐26b‐5p/QKI‐5 axis formed a feedback loop to regulate proliferation and tube formation of HUVECs

**Figure 8:**
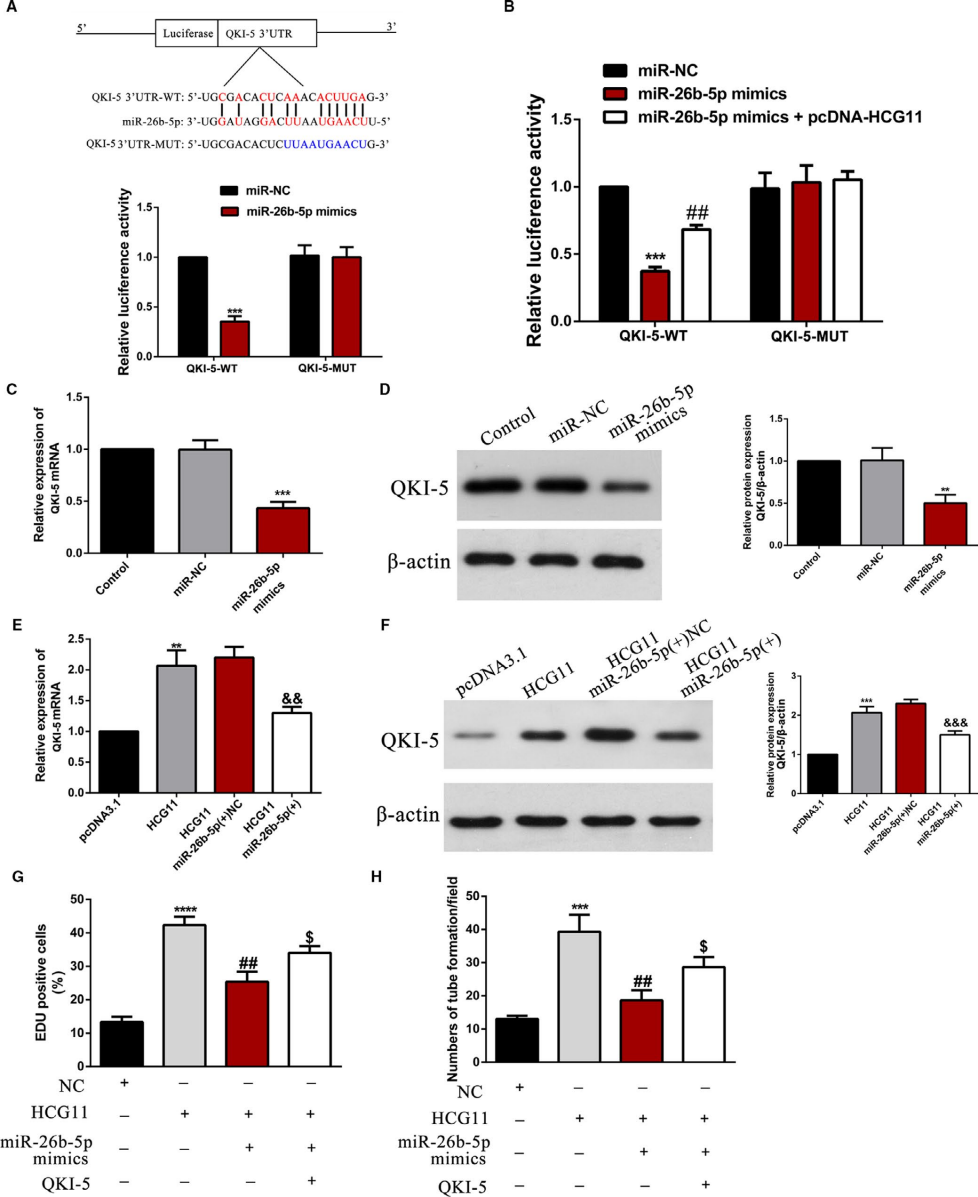
LncRNA HCG11/miR‐26b‐5p/QKI‐5 feedback loop regulates proliferation and tube formation of HUVECs. (A‐B) Schematic representation of the putative miR‐26b‐5p binding sites in QKI‐5, and the relative luciferase activity was detected in HUVECs cotransfected with wild‐type or mutated QKI‐5 reporter plasmid together with miR‐26b‐5p mimics or miR‐26b‐5p plus lncRNA HCG11 overexpression plasmid, ^***^ *P* < .001 vsmiR‐NC group, ^##^ *P* < .01 vsmiR‐26b‐5p mimics group. (C‐D) The effect of miR‐26b‐5p on the expression of QKI‐5 was detected by qRT‐PCR and western blot, ^***^ *P* < .001, ^**^ *P* < .01 vs. miR‐NC group. (E‐F) The mRNA and protein expression levels were regulated by lncRNA HCG11 and miR‐26b‐5p, ^**^ *P* < .01 vs. PcDNA3.1, ^&&^ *P* < .01,^&&&^ *P* < .001 vs. LncRNA HCG11 + miR‐26b‐5p(+)NC group. (G‐H) The co‐effect of lncRNA HCG11, miR‐26b‐5p and QKI‐5 on the proliferation and tube formation of HUVECs. ^***^ *P* < .001,^****^ *P* < .0001 vs. NC group; ^##^ *P* < .01 vs. HCG11 group; ^&^ *P* < .05 vs. MiR‐26b‐5p group

StarBase analysis indicated that QKI‐5 was predicted to be a target gene of miR‐26b‐5p. The predicted combination sequence of miR‐26b‐5p on QKI‐5 are shown (Figure 8A). The results of dual‐luciferase reporter assays demonstrated that cotransfection with wild‐type QKI‐5 (QKI‐5‐WT) plasmids and miR‐26b‐5p mimics remarkably reduced the luciferase activities of HUVECs (*P* < .001), whereas the luciferase activities were not changed when the cells were cotransfected with mutant QKI‐5 (QKI‐5‐MUT) plasmids as well as miR‐26b‐5p mimics (Figure 8A), suggesting the interaction between miR‐26b‐5p and QKI‐5. Furthermore, we cotransfected with pcDNA‐HCG11 in both groups, the former effect of the luciferase activities was partly reversed and the latter did not (Figure 8B). Deservedly, both the mRNA and protein expression level of QKI‐5 were decreased after up‐regulation of miR‐26b‐5p (Figure 8C‐D). While this inhibition effect was impaired in response to overexpression of lncRNA HCG11 (Figure 8E‐F). For function assay, the proliferation and angiogenesis promoting of lncRNA HCG11 overexpression were reduced by miR‐26b‐5p, but partially rescued by the up‐regulation of QKI‐5 (Figure 8G‐H), Collectively, lncRNA HCG11 promoted HUVECs proliferation and angiogenesis through regulating miR‐26b‐5p/QKI‐5 feedback loop axis.

## 4 DISCUSSION

Recent years lncRNAs have been implicated as potential biomarkers in the progression of antherosclerosis.^25^, ^26^, ^27^ For instance, lncRNA MIAT aggravates atherosclerotic damage via activation PI3K/Akt signalling pathway,^10^ LncRNA MANTIS played an crucial role in the beneficial effects of stain treatment and laminar flow^28^; LncRNA CDKN2B‐AS1 decreased inflammatory response and promoted cholesterol efflux through inhibited the transcription of ADAM10.^29^ This research indicated lncRNAs played an important role in the development of antherosclerosis. Our study first exhibited high expression of lncRNA HCG11 in patients of vulnerable plaque and investigated the function of LncRNA HCG11 in HG‐induced HUVECs. Functionally, we found lncRNA HCG11 was reduced in HG‐treated HUVECs, and overexpression of lncRNA HCG11 rescued HG‐induced injury of HUVECs. Furthermore, we demonstrated QKI‐5 is a downstream target of lncRNA HCG11, meanwhile, QKI‐5 as a RNA‐binding protein combined with HCG11 promoted stability of lncRNA HCG11, all these experiments results suggested that lncRNA HCG11 played proliferation and angiogenesis promotion role in HG‐treated HUVECs.

High FBG has long been taken into account as a risk factor for endothelial cell dysfunction and death in atherosclerosis. Many studies have found that high concentrations of glucose injured ECs angiogenesis.^30^, ^31^, ^32^ Our present study verified that high concentrations of glucose (11 and 33 mg/ml) reduced the proliferation and tube formation of HUVECs in a time‐dependant and dose‐dependant manner (Figure 2E‐F). These findings were consistent with previous reports. LncRNA HCG11 was reduced in HG‐treated HUVECs, overexpression of lncRNA HCG11 reversed the proliferation and tube formation inhibition of HG treatment, so this indicated lncRNA HCG11 played an important role in the mechanism of HG‐induced ECs injury.

In general, lncRNA worked as competing endogenous RNAs (ceRNA) to regulate miRNA‐mRNA axis. Yet there is increasing evidence indicate that RNA‐binding proteins can affect biological functions of cells through regulating the stability of lncRNAs. Such as, UPF1 regulates the progression of glioblastoma cells by improving the stability of linc‐00313,^33^ SRSF1 stabilized lncRNA NEAT1 to regulate the cell cycle progression in glioma.^34^ Transcript factors Nanog, Sox2 and Fgf4 promote neural differentiation of mouse ESCs through binding with lncRNA TUNA.^35^ In our study, experiments suggested HCG11 as a ceRNA competing binding with miR‐26b‐5p increased the transcripts of QKI‐5, meanwhile, QKI‐5 increased lncRNA HCG11 expression through directly binding with LncRNA HCG11.

Relevant reports indicated that QKI‐5 promotes angiogenesis of ECs through directly binding with STAT3 3’UTR and stabilized STAT3 mRNA.^22^ In heart failure process, QKI‐5 improved cardiac function by combined with circRNAS Ttn, Fhod3 and Strn3.^36^ In our study, the binding site of QKI‐5 and lncRNA HCG11 was predicted with catRAPID. According to the prediction, RIP assay was performed to verified that QKI‐5 could combined with lncRNA HCG11. And then, the combination increased the expression of lncRNA HCG11 promoted the proliferation and angiogenesis of HUVECs. It was further confirmed that QKI silencing significantly decreased the expression of lncRNA HCG11. Based on our available results, it is an attractive possibility that QKI‐5 specific interacted with lncRNA HCG11 and promote lncRNA HCG11 expression through increased the stability of lncRNA HCG11. Nevertheless, there is reported that QKI‐5 is regulated by interacted with lnc10,^37^ so, the interaction of lncRNA and RBP can effect functions of both lncRNA and RBP. In this study, we identified QKI‐5 can be regulated by lncRNA HCG11, but it is not enough to prove this regulator is through direct interaction, this still needs further research.

LncRNAs play their roles through many mechanisms including transcriptional and post‐transcriptional regulation, miRNA sponges, mRNA binders and protein binders.^38^ Our research demonstrated that miR‐26b‐5p was down‐regulated in patients with vulnerable plaque and high expressed in HG‐induced endothelial injury. Down‐regulated of miR‐26b‐5p partly reversed HG‐induced proliferation and tube formation inhibition of HUVECs. Our findings are consistent with other research teams. MiR‐26b‐5p inhibited angiogenesis and fibrogenesis of mouse liver through targeting PDGF receptor‐β^39^; MiR‐26b‐5p suppressed vascular mimicry and angiogenesis through depressed the expression of MMP2, VE‐cadherin and snail.^20^ The bioinformatic analysis found binding sites between lncRNA HCG11 and miR‐26b‐5p, suggesting that lncRNA HCG11 functioned as a miR‐26b‐5p sponge to regulated physiological function of endothelial cells. Further experiments proved that overexpression of lncRNA HCG11 reduced miR‐26b‐5p, meanwhile, overexpression of miR‐26b‐5p blocked lncRNA HCG11 induced proliferation and tube formation of HUVECs. Through dual‐luciferase reporter assay, there is an inhibitory relationship between HCG11 and miR‐26b‐5p. LncRNA worked as a miRNA sponges is very common and prevalent in different physical or disease process. Such as, lncRNA HCG11 regulated glioma cell progression through cooperating with miR‐496/CPEB3 axis^40^; LncRNA LINC00657 promoted NSCLS by regulating miR‐26b‐5p/COMMD8 axis.^41^

miRNAs usually repressed gene expression by combined with 3’UTR of specific mRNA, in our study, bioinformatics predicted miR‐26b‐5p might bind to the position of 017‐022 in QKI‐5 3’‐UTR. Further reporter vectors construction and dual‐luciferase reporter were implemented to verify the binding sites between miR‐26b‐5p and QKI‐5, respectively. In HG‐treated HUVECs, overexpression of lncRNA HCG11 significantly increased the expression of QKI‐5. While gain or out of miR‐26b‐5p obviously reduced or increased QKI‐5 expression. Functional experiments showed that, overexpression of QKI‐5 increased the proliferation and tube formation of HUVECs, and lncRNA HCG11 induced cell proliferation could be inhibited by overexpression of miR‐26b‐5p or knockdown of QKI‐5. In this study, we found that the combined sequence (AAUGAACU) between miR‐26b‐5p and lncRNA HCG11 is much of the same as the bind to the QKI‐5 3’UTR. Above‐mentioned results indicated that lncRNA HCG11 worked as a ceRNA binding with miR‐26b‐5p, and then attenuated the inhibitory effect of miR‐26b‐5p on QKI‐5 3’UTR, finally affected the progression of HUVECs.

Interestingly, the combination between QKI‐5 and lncRNA HCG11 is predicted by catRAPID and confirmed by RIP assay. Overexpression of QKI‐5 increased the expression of lncRNA HCG11, simultaneously, lncRNA HCG11 sponged miR‐26b‐5p and reduced the suppression of miR‐26b‐5p on QKI‐5 3’UTR. Taking together, these results suggested that lncRNA HCG11/miR‐26b‐5p/QKI‐5 formed a positive‐feedback loop to regulate the physiological function of HUVECs (Supplementary Figure 2). These findings provide new insights for the diagnose of the vulnerable plaque, as well as a new therapeutic target for the treatment of AS in the future.

## 5 CONCLUSION

Our study revered that the feedback loop of lncRNA HCG11/miR‐26b‐5p/QKI‐5 played a vital role in the physiological function of HUVECs and that could provide a potential target for therapeutic strategies of As.

## CONFLICT OF INTEREST

We declare that the authors have no competing interests that might be perceived to influence the results and/or discussion reported in this paper.

## AUTHOR CONTRIBUTION

**Jiao Du:** Data curation (equal); Investigation (equal); Writing‐original draft (equal). **Ruijuan Han:** Methodology (equal); Project administration (equal). **Yihua Li:** Data curation (equal); Formal analysis (equal). **Xiaolin Liu:** Software (equal). **Shurong Liu:** Investigation (equal). **Zhenyu Cai:** Methodology (equal). **Zhaolong Xu:** Writing‐review & editing (equal). **Ya Li:** Writing‐review & editing (equal). **Xuchun Yuan:** Writing‐review & editing (equal). **Kai Sun:** Conceptualization (equal); Funding acquisition (equal). **Xiuhai Guo:** Conceptualization (equal). **Bin lu:** Conceptualization (equal); Funding acquisition (equal).

## Supporting information

Fig S1

Fig S2
