# Supplementary figures and images for: LncRNA HCG11/miR‐26b‐5p/QKI5 feedback loop reversed high glucose‐induced proliferation and angiogenesis inhibition of HUVECs
Source: J Cell Mol Med. 2020 Oct 30;24(24):14231–46. doi: 10.1111/jcmm.16040 (PMC7753996; doi:10.1111/jcmm.16040)

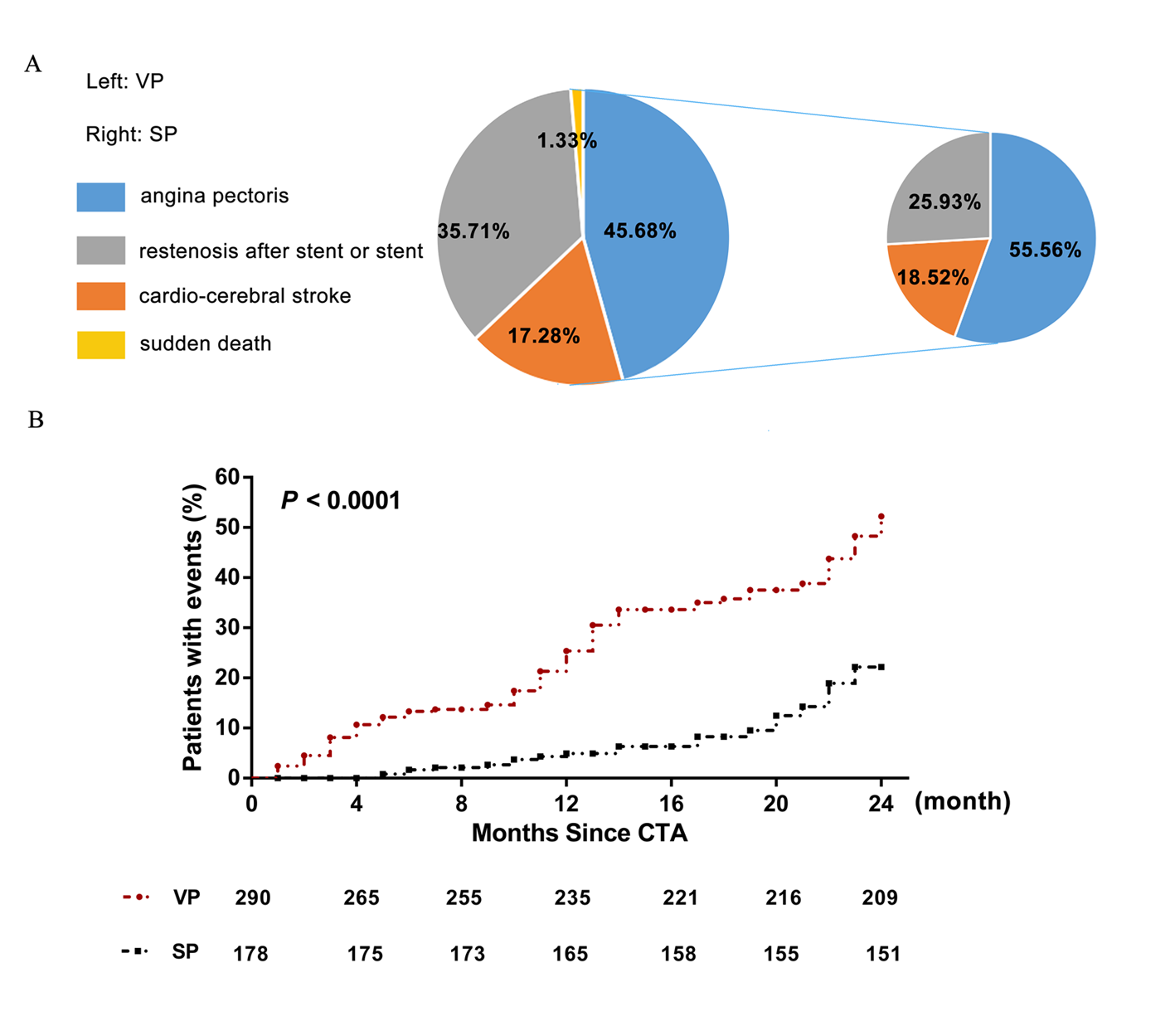

Supplement: Supplementary file 1 — Fig S1 [file JCMM-24-14231-s001.tif]

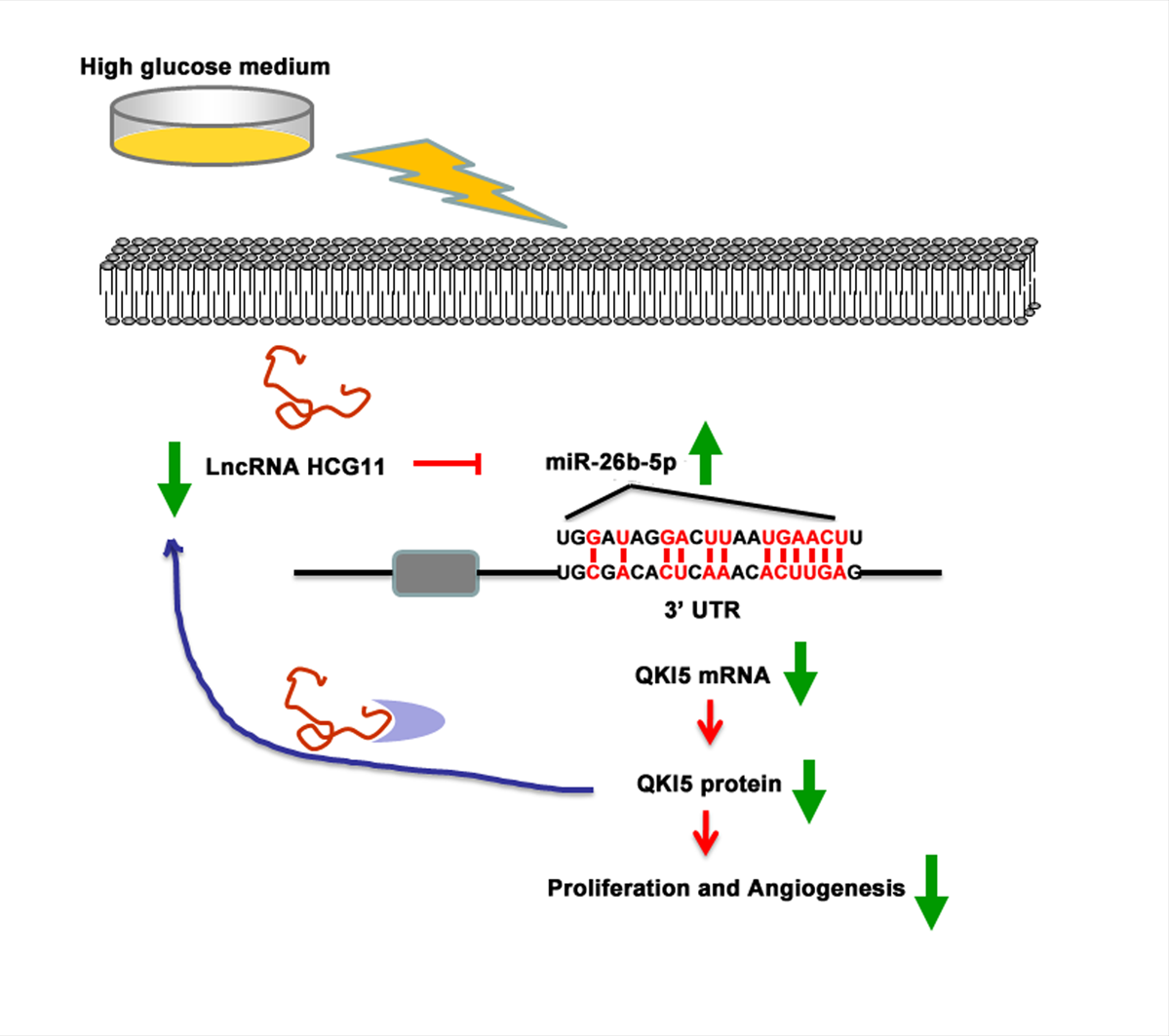

Supplement: Supplementary file 2 — Fig S2 [file JCMM-24-14231-s002.tif]
